# Supplementary material for: Health professionals’ perceptions on local production and bioequivalence study of generic medicines: A cross-sectional survey of physicians and pharmacy professionals in Addis Ababa, Ethiopia
Source: PLoS One. 2023 Mar 27;18(3):e0281665. doi: 10.1371/journal.pone.0281665 (PMC10042336; doi:10.1371/journal.pone.0281665)
Supplement: S1 File — (DOCX) [file pone.0281665.s001.docx]

**Addis Ababa University**

**College of Health Sciences**

**School of Pharmacy**

**Department of Pharmaceutics and Social Pharmacy**

**Self-administered Questionnaire**

**Introduction:**

Attached herewith is a self-administered questionnaire developed to identify the perception of health professionals and policy makers towards local production and bioequivalence **(BE)** studies for generic medicines. The research is also aimed at investigating the opportunities and barriers to local pharmaceutical plants in carrying out BE studies. This study is conducted by AAU, College of Health Sciences, School of Pharmacy, Department of Pharmaceutics and Social Pharmacy.

Your participation is purely voluntary and information you provide will be kept completely confidential. Your name will never be written and aggregate responses from different respondents are identified only by codes.

Your honest response to the questions is of paramount importance for the successful completion of the study. You can have clarification for any doubt regarding the questions. If you have any questions or comment in the meantime, please feel free to contact the Co-PI, Muluken Nigatu (Tel: 0912159807) and AbrhamTemesgen (Tel: 0913381523).

Are you willing to respond to the questions? Yes 🞏 No 🞏

If your answer is yes, go to the next page for the questions.

**Part one: Socio-demographic characteristic of respondents**

**Direction**: please put /**🗸**/ mark in the box provided or write your response on space provided. Answer all the items which apply to you.

- 1. Age:_____ years
  2. Gender: 🞏 Male 🞏 Female
  3. Highest qualification you obtained:

🞏 General practitioner (GP) 🞏 Specialty 🞏 Druggist 🞏 BPharm

🞏 MSc in Pharmacy 🞏 Others, specify: ___________________________

- 1. Area of current practice?

🞏 Hospital 🞏 Community pharmacy 🞏 Pharmaceutical industry

🞏 FBPIDI 🞏 EFMHACA 🞏 PFSA 🞏 FMOH 🞏 Others, specify____________

- 1. Total years of work experience (in current area of practice): ___________years

**Part two: Perception/awareness of respondents towards BE study**

- 1. When you want to prescribe/purchase medicines, which one do you prefer?

🞏 Locally manufactured products 🞏 Imported products 🞏 No preference

- 1. If you have specific preference to Question #1, what is/are the reason (s) for your preference (more than one answer is possible)?

🞏 Cheap price 🞏 Easily available 🞏 Better quality 🞏 More effective

🞏 Well promoted 🞏 Others, specify_____________

- 1. If your answer for Question # 1 is ‘imported products’, which country of origin do you prefer most? (more than one answer is possible)

🞏 China 🞏 Germany 🞏 India 🞏 Cyprus 🞏 Other, specify_________

- 1. Have you heard about the term Bioequivalence? 🞏 Yes 🞏 No

* EFMHACA = Ethiopian Food, Medicines and Health Care Administration and Control Authority

*FBPIDI = Food, Beverage and Pharmaceutical Industries Development Institute

*FMOH = Federal Ministry of Health

*PFSA = Pharmaceutical Fund Supply Agency

- 1. To be called Bioequivalent, products must have/show? (more than one answer is possible)

🞏 Same drugs with same price 🞏 Same drugs with the same strength

🞏 Same drugs with similar safety and efficacy 🞏 Same drugs with the same dosage form

🞏 Other, Specify_________________

- 1. Is there bioequivalence test center in Ethiopia? 🞏 Yes 🞏 No 🞏 Unsure
  2. Do you think that all immediate release pharmaceutical products require BE study for registration by EFMHACA?

🞏 Yes 🞏 No 🞏 Unsure

- 1. For which dosage forms is BE study necessary? (more than one answer is possible)

🞏 Tablet 🞏 Capsule 🞏 Suspension 🞏 Syrup 🞏 Other, specify ______

- 1. Do you think that local pharmaceutical plants implement BE studies?

🞏 Yes 🞏 No 🞏 Unsure

- 1. If your answer is **No** for Question #9, what do you think the reason for local pharmaceutical plants not to carry out BE studies? (More than one answer is possible)

🞏 BE centers out of reach 🞏 BE centers' price is unaffordable

🞏 Regulatory authority does not enforce companies to undertake BE studies

🞏 Less commitment from the company owner/management

🞏 Long waiting time for results

🞏 Other, specify __________________________________

- 1. Do you think EFMHACA should implement enforcement of BE requirements for locally manufactured immediate release products?

🞏 Yes 🞏 No 🞏 Unsure

- 1. If your answer to Question 11 is '**Yes**', where should BE studies be conducted?

🞏 Locally 🞏 Abroad 🞏 Both locally and abroad

- 1. Do you think there is apparent advantage of conducting BE locally?

🞏 Yes 🞏 No 🞏 Unsure

- 1. If your answer to Question 13 is '**Yes**', what do you think are the advantages? (more than one answer is possible)

🞏 BE price will be affordable

🞏 BE center will be more accessible

🞏 BE results will be more reliable/dependable

🞏 Shorter waiting time for results

🞏 Other, specify __________________________________

If you have further points or comments to add, we will be appreciating.

________________________________________________________________________________________________________________________________________________________________________________________________________________________

**Thank you!!!**
